# Hair Cortisol Research in Posttraumatic Stress Disorder - 10 Years of Insights and Open Questions. A Systematic Review

**DOI:** 10.2174/1570159X21666230807112425

**Published:** 2023-08-07

**Authors:** Lena Schindler-Gmelch, Klara Capito, Susann Steudte-Schmiedgen, Clemens Kirschbaum, Matthias Berking

**Affiliations:** 1 Department of Clinical Psychology and Psychotherapy, Friedrich-Alexander-Universität Erlangen-Nürnberg, Erlangen, Germany;; 2 Department of Psychotherapy and Psychosomatic Medicine, Faculty of Medicine, Technische Universität Dresden, Dresden, Germany;; 3 Faculty of Psychology, Technische Universität Dresden, Dresden, Germany

**Keywords:** Posttraumatic stress disorder, trauma, hair cortisol, diagnostic, prognostic, intervention-related, biomarker

## Abstract

**Background:**

Cortisol is one of the most extensively studied biomarkers in the context of trauma/posttraumatic stress disorder (PTSD). For more than a decade, hair cortisol concentrations (HCC) have been measured in this context, leading to a two-staged dysregulation model. Specifically, an elevated secretion during/immediately after trauma exposure eventually reverts to hyposecretion with increasing time since trauma exposure has been postulated.

**Objective:**

The aim of our systematic review was to re-evaluate the two-staged secretion model with regard to the accumulated diagnostic, prognostic, and intervention-related evidence of HCC in lifetime trauma exposure and PTSD. Further, we provide an overview of open questions, particularly with respect to reporting standards and quality criteria.

**Methods:**

A systematic literature search yielded 5,046 records, of which 31 studies were included.

**Results:**

For recent/ongoing (traumatic) stress, the predictions of cortisol hypersecretion could be largely confirmed. However, for the assumed hyposecretion temporally more distal to trauma exposure, the results are more ambiguous. As most studies did not report holistic overviews of trauma history and confounding influences, this may largely be attributable to methodological limitations. Data on the prognostic and intervention-related benefits of HCC remain sparse.

**Conclusion:**

Over the last decade, important insights could be gained about long-term cortisol secretion patterns following lifetime trauma exposure and PTSD. This systematic review integrates these insights into an updated secretion model for trauma/PTSD. We conclude with recommendations for improving HCC research in the context of trauma/PTSD in order to answer the remaining open questions.

## 1 INTRODUCTION

Posttraumatic stress disorder (PTSD) is a stressor-related psychiatric disorder that can occur following exposure to a traumatic event, characterized by actual or threatened death, serious injury, or sexual violence [1]. The core symptoms include intrusions, avoidance of internal and external reminders of the traumatic event(s), hyperarousal, and negative alterations of cognition and mood.

Among the most extensively studied underlying biological mechanisms of PTSD are dysregulations of the hypothalamic-pituitary-adrenal (HPA) axis and its central effector hormone cortisol [2-4]. The HPA axis is a neuroendocrine system of complex hormonal cascades following a circadian rhythm, but also activated in response to psychological or physiological stress [5, 6]. Its postulated main function is the maintenance of homeostasis in the face of perpetually changing intrinsic and extrinsic demands [5, 6]. Physical stressors (*e.g*., pain, noise) are mainly processed in the brain stem (*i.e*., the nucleus of the solitary tract), and dorsomedial hypothalamus, and psychological stressors (*e.g*., perceived stress, anxiety, fear) are mainly processed in the limbic system (*i.e*., the amygdala and prefrontal cortex areas) leading to an activation of the nucleus paraventricularis (PVN) of the hypothalamus. In response, corticotropin-releasing hormone (CRH) and arginine-vasopressin (AVP) are released, stimulating the secretion of adrenocorticotropin-releasing hormone (ACTH) from the pituitary. ACTH reaches the adrenal glands through the blood stream, where glucocorticoid hormones such as cortisol are released from the zona fasciculata of the adrenal cortex. By binding on glucocorticoid and mineralocorticoid receptors, its free, unbound fraction reaches the central nervous system (CNS) as well as peripheral organs, and effects can occur within a time frame of minutes to hours due to a mostly genomic pathway [5-7]. Cortisol plays a crucial role in providing energy for coping with tasks and/ or stressors [8, 9], by enhancing glucose production, suppressing the immune system, or influencing the secretion of further hormonal or neurotransmitter agents associated with reward processing, attention, executive functioning, and emotion [7, 9]. Under conditions of repeated, prolonged, and intense secretion, cortisol may also have adverse sequelae, such as neurotoxicity, maladaptive neuronal alterations, and impairments of the immune system [7]. Notably, due to negative feedback loops, the HPA axis may become dysregulated both towards hyper- and towards hypo-activity following certain severe types of strain, in which both are assumed to be accompanied by distinct sequelae [5]. It has been frequently postulated that core symptoms of PTSD such as altered memory for trauma-associated stimuli (intrusive memories on the one hand and partial amnesia for the traumatic event on the other), changes in mood, or hyperarousal, may be at least partially explained by such changes of HPA axis functioning [10].

Cortisol is typically assessed *via* blood, saliva, and urine sampling. These methods have led to important insights, such as the role of trauma exposure, and not only clinically relevant PTSD, for cortisol alterations, the influence of time since trauma exposure, and the relevance of cortisol alterations as both sequelae and risk factors for PTSD [11, 12]. However, they only allow insights into time frames of seconds to hours [13, 14]. Thus, discrepant study results in trauma or PTSD research (*i.e*., studies reporting higher, lower, or unchanged cortisol secretion compared to trauma-exposed or non-exposed controls, for meta-analytic data see, *e.g*., 2, 11, 12, 15) were often attributed to this methodological aspect. In particular, the sensitivity of such methods to cortisol’s circadian rhythm and situational (and thus, potentially confounding) influences are limiting factors when studying long-term psychological conditions such as those following trauma exposure [3]. Thus, the methodological break-through of applying hair analyses for psychoneuroendocrine research [13] elicited high hopes of finally being able to solve the puzzle of hyper- *versus* hypocortisolism findings in trauma/PTSD. Due to a postulated hair growth rate of approximately 1 cm per month [16, 17], the scalp-near 1 cm hair segment has previously been assumed to retrospectively reflect cumulative cortisol secretion over a period of one month [14]. However, recent findings from animal models rather suggest glucocorticoids to be integrated into and diffuse out of the hair in an ongoing fashion even after the hair has grown out of the follicle [18]. If confirmed, hair cortisol concentration (HCC) would need to be interpreted as a marker of current (albeit long-term) stress rather than a “calendar-like” evidence for past stressors. Nevertheless, in human samples, HCC has been validated with accumulated cortisol levels derived from saliva and urine samples and shown to have high retest reliability under stable environmental conditions [13, 19]. As such, it can currently be considered an apt way to non-invasively study longer-term endocrine secretion with minimal burden to participants and requirements of laboratory settings (*e.g*., no cooling, handling of potentially infectious material, or medical training for venipuncture required).

First results in trauma/PTSD have been promising, leading to a preliminary dose- and time-dependent model of cortisol secretion [4]. In general, a two-staged trajectory has been proposed, mirroring the one postulated for chronic stress conditions in general [20-22]. In detail, the model predicts elevated cortisol secretion immediately after trauma exposure, which then - possibly due to dysregulated negative feedback loops - reverts to an attenuated cortisol secretion as time since the traumatic event increases [4]. Further, a dose-dependent endocrine “building block effect” has been hypothesized, mirroring the clinical observation of a higher risk for more severe PTSD trajectories with increasing trauma load on an endocrine level. Thus, alterations of cortisol secretion were assumed to not only be trauma sequelae, but also risk factors for the development of PTSD [4], as well as important mechanisms for the monitoring and enhancement of PTSD-related therapy. Importantly, the model was based on a total of only *n* = 8 predominantly cross-sectional HCC studies available at the time of publication.

In recent years, several systematic reviews and meta-analyses have reported on cortisol in trauma/PTSD research. However, due to their time of publication, a deviating focus and/or strict inclusion criteria, the majority reported on few if any HCC studies [*e.g*., *n* = 1; 2, *n* = 4; 21, *n* = 0; 22]. Further, many [23, 24] exclusively or predominantly focused on childhood adversity (*i.e*., emotional, physical, or sexual abuse as well as emotional or physical neglect) as typically assessed *via* the Childhood Trauma Questionnaire [25]. Although such experiences undisputedly lead to a higher risk of psychopathology over the lifespan [26] and a certain overlap exists, not every type of childhood adversity, or adversity in general, automatically qualifies as a traumatic event according to DSM-IV/DSM-5 [1, 27]. For instance, emotional neglect would qualify as the first, but not the latter due to not being “exposure to actual or threatened death, serious injury, or sexual violence” [1]. While cortisol secretion in the context of (childhood) adversity has recently been exhaustively reviewed [23, 24], a current overview of studies focusing on lifetime trauma exposure (LTE) as defined by DSM-IV/DSM-5 is still pending.

Thus, the current study aimed to provide a systematic, topical overview of the literature on HCC in the context of LTE and PTSD as defined by DSM-IV/DSM-5 criteria as an update of the model by Steudte-Schmiedgen and colleagues [4]. For this, we followed the suggested framework by Engel *et al.* [3] and categorized the available data as diagnostic (*i.e*., HCC utilized to discern individuals with and without trauma exposure/PTSD), prognostic (*i.e*., HCC utilized to predict subsequent symptom trajectories), and intervention-related (*i.e*., HCC utilized to study psychotherapeutic outcome). Further, we intended to provide insights into the reporting standards/quality of the available literature regarding typical confounders for HCC data. The study follows the recommendations of the Preferred Reporting Items for Systematic Reviews and Meta-Analyses (PRISMA) group [28]. It is part of a larger preregistration for a systematic review and meta-analysis at PROSPERO on July 20, 2022 (registration number: CRD42022344274).

## 2 MATERIALS AND METHODS

### 2.1 Inclusion Criteria

Studies were included in the systematic review if they met the following criteria: 1) Cortisol was assessed at one or more time points using scalp hair samples and was analyzed to determine its association with trauma status or PTSD symptomatology. 2) Trauma status and/or PTSD symptomatology according to DSM-IV/DSM-5 criteria [1, 27] were assessed with a state-of-the-art self-report clinical interview or questionnaire. The following exclusion criteria were established: studies 1) were published prior to 2004, when Raul, Cirimele, Ludes, and Kintz [29, 30] described HCC analyses in humans for the first time. 2) did not provide full-text in either English or German. 3) did not report on human, living participants. 4) did not report primary research (*e.g*., reviews, meta-analyses, expert opinions, study protocols, *etc*.). 5) assessed children/adolescents below the age of 18 years. and 6) had not been published in a peer-reviewed journal.

For the full-text screening, in accordance with the specific focus of the current systematic review, the following additional criteria led to an exclusion: studies 7) were conducted during pregnancy or the post-partum period (in order to account for the respective hormonal changes, *e.g*., 30). or 8) did not allow a differentiation between LTE and (childhood) adversity. If studies reported on samples including both adults and children/adolescents, they were excluded if no separate analyses for adults had been conducted, with the exception of one study which clearly reported that only two of 64 participants were younger than 18 years [31]. In cases of separate analyses, only the data on the adult subsample were included. To the best of our knowledge, if more than one published manuscript was based on the same sample, the one with the bigger data set was used in order to both avoid overlaps and increase statistical power. During the literature search, it emerged that two of the included studies [32, 33] had utilized a singular hair sample for longitudinal assessments (by analyzing separate segments for chronological insights into cortisol secretion). Due to the relevant concerns discussed, *e.g*., by Kalliokoski, Jellestad, & Murison [34] and Stalder *et al.* [19], only cross-sectional, but not longitudinal analyses are reported for those studies.

### 2.2 Identification and Selection of Studies

The literature search was conducted as recommended by Cuijpers [35]. We conducted a full-text/all-fields search in the databases Pubmed, Scopus, Web of Science, Medline, PsycInfo, and PsycArticles on July 20, 2022 with the search terms ”trauma*” OR ”posttraumatic” OR ”PTSD” AND ”cortisol” AND ”hair” (see Appendix A for the exact search terms). In addition, a snowball search system was used to detect additional potentially relevant studies by screening the reference lists of relevant systematic reviews/meta-analyses [2, 4, 21, 24] and included studies. Data management was conducted *via* Rayyan [36]. Two authors (LSG, KC) decided on the inclusion or exclusion of each study. In the beginning, *n* = 30 abstracts were screened and discussed together as recommended by Cuijpers [35]. Another meeting was scheduled after ~10% of abstracts were screened to discuss potential special cases. After that, bi-weekly meetings were conducted to counteract coder drift during abstract or full-text screening. In case of disagreement, the consensus was reached *via* discussion. The percentage of agreement was calculated. Fig. (**1**) provides a flowchart for study selection.

### 2.3 Data Extraction and Coding of Study Characteristics

Data were extracted by LSG and cross-checked by KC as well as two student research assistants. Tables **1-5** summarize the extracted data as well as the characteristics of the included studies.

### 2.4 Assessment of Reporting Standards and Study Quality

An assessment of study quality and reporting standards was conducted with a self-developed checklist closely based on the CoAL checklist referring to covariates caused by the sampling design or the assessed individual [37]. As the tool is currently available only for blood, saliva, or urine cortisol assessments, it was adapted for hair sampling following empirical insights on potential confounding variables [14, 19, 38] as well as the suggested quality criteria from similar systematic works [23]. The criteria for reporting standards were grouped into five categories for descriptive purposes: 1) trait characteristics of the participant (age, sex, body mass index, socioeconomic status, ethnicity). 2) substance and medication intake of the participant (nicotine, alcohol, drugs, hormonal contraceptives, overall, psychotropic, endocrine, specifically glucocorticoid-containing medication). 3) health-related characteristics of the participant (presence of severe/ chronic physical or psychological conditions, specifically endocrine disorders, pregnancy, lactation/breastfeeding, menopause, major rhythm changes, subjectively experienced stress). 4) hair characteristics (natural color, curls/waves, washing frequency, hair treatments). and 5) hair sampling and analysis factors (season of sampling, sampled at posterior vertex length ≤ 6 cm, hair mass, storage time, analysis in one batch, inter- and intra-assay coefficients of variance, non-detectables and outliers, corrections for skewness). Further, exact details on trauma/PTSD (*i.e*., type of focus trauma, assessment of PTSD symptomatology, assessment of number and timing of LTE) were extracted and reported. As the CoAL manual suggests to individually adapt criteria depending on the study context, we decided to rate for confounders as follows: *did not report* = 0; *did report* = 1; or *did control for a confounder* (*e.g*., by demonstrating no respective group differences, calculating its association with HCC, adding it as a covariate, or excluding it/fixing it to a certain value/imputing it) = 2. We calculated sums, mean scores, and standard deviations for each of the five categories: 1) Five items corresponding to a range of 0-10; 2) eight items corresponding to a range of 0-16; 3) eight items corresponding to a range of 0-16; 4) four items corresponding to a range of 0-8; 5) 11 items corresponding to a range of 0-22. The resulting mean scores were then rated as- -(x < 0.2, no to minimal reporting), - (0.2 ≤ x < 0.4, poor reporting), = (0.4 ≤ x < 0.6, average reporting), + (0.6 ≤ x < 0.8, good reporting), and ++ (0.8 ≤ x ≤ 1, very good to excellent reporting). The full checklist applied is available in Appendix B. Study quality was assessed by LSG and KC independently, with two student research assistants independently crosschecking results. Again, in case of disagreement, the consensus was reached *via* discussion.

## 3 RESULTS

### 3.1 Included Studies

From the 5,046 studies identified in the first search, 31 studies (corresponding to *n* = 3,576 participants) were included in the systematic review. The percentage of initial agreement between coders was 95.4% for the abstract, and 89.4% for the full-text screening.

### 3.2 HCC as a Diagnostic Biomarker in Trauma/PTSD

Fifteen of the identified studies reported on HCC group differences in the context of trauma/PTSD (Table **1**). Among those, nine studies directly contrasted PTSD and/or trauma-exposed (TE) and/or non-trauma-exposed (NTE) groups. Four reported elevated HCC in individuals with a PTSD diagnosis compared to TE [39, 40] or NTE controls [41, 42], albeit the latter at trend level. One found elevated HCC in a TE compared to an NTE group [43]. In contrast, three studies reported lower HCC compared to TE [44] or NTE controls [45, 46], albeit the latter at trend level, and one study showed no group differences between PTSD and TE group [47]. When taking a closer look at the pattern of findings, it became evident that all studies reporting on relatively recent trauma exposure or individuals still facing high-stress living conditions found elevated HCC compared to respective control groups [39, 40, 43 and 42, albeit the latter at trend level]. With regard to studies on temporally more distal trauma exposure (*i.e*., several years since the subjectively worst event) or not reporting at least a rough estimate of the time since trauma exposure, the picture emerged as far less clear, with higher [41], lower [44, 45, 46, albeit the latter at trend level] or no differences in HCC [47] reported compared to respective control groups. Further, it emerged that out of the six studies directly contrasting PTSD and TE groups, three yielded no group difference [41, 45, 47; but not 39, 40, 44]. In contrast, all four studies comparing PTSD or PTSD/TE with NTE groups found group differences [41, 42, 45, 46]; albeit 42 and 46 at trend level, respectively.

In addition, six studies focused on specific subtypes of trauma exposure (*e.g*., individuals with or without exposure to interpersonal violence). The picture here mirrored the results from above in that the studies reporting elevated HCC as compared to controls exposed or non-exposed to the respective event predominantly focused on more recent or ongoing events [48-50; but 51]. In contrast, one study reporting childhood sexual abuse conducted roughly 40 years ago found lower HCC in the exposed compared to the non-exposed group [52]. Lastly, one study contrasting individuals with both childhood trauma and recent exposure to violence with individuals with childhood trauma, but no recent exposure to violence, as well as non-exposed controls, did not find any group differences [53].

As reported in Table **2**, 27 studies reported associations of HCC with indicators of trauma and/or PTSD. Importantly, no study reported contradicting group-level and associative findings (*e.g*., lower HCC in PTSD compared to NTE individuals, but positive associations with symptom severity). Seventeen studies focused on PTSD symptomatology, with four reporting positive [40, 54-56], two negative [45, 57], and twelve no significant associations. Importantly, all four studies reporting positive associations focused on samples with recent or ongoing trauma exposure. It was not possible to identify symptom clusters particularly closely associated with HCC. The few studies which reported any associations found those for hyperarousal [55], intrusions [40, 45], and changes in mood/cognition [40]. Three studies reported associations of HCC with the time since trauma exposure, with one reporting positive [50, for individuals with recent exposure, *i.e*., within the last 12 months], one negative [45, with 72% of the participants reporting > 5 years since trauma exposure] and one no associations [51, with 85.5% of the participants reporting ≥ 10 years since last trauma exposure]. Among the 17 studies researching indices of LTE (*e.g*., yes/no, number of different traumatic events, total frequency of trauma exposure), four were majorly positive [39, 40, 47, 58], three negative [45, 46, 59], and seven had no associations [31, 56, 60-64]. Three studies found different associations for different subtypes of trauma exposure. Fischer *et al.* [65] reported positive associations with war exposure (yes/no), negative ones with crime victimization (yes/no), and overall no associations with the total number of different events, while Andersen *et al.* [59] found no associations with the number of prior interpersonal trauma events, but inverse ones with the number of prior bereavement events. Further, Castro-Vale *et al.* [47] found associations with the number of types of war exposure only in individuals without lifetime major depressive disorder.

### 3.3 HCC as a Prognostic Biomarker in Trauma/PTSD

Four studies utilized HCC to predict symptom trajectories in the context of trauma/PTSD. However, their study designs differed markedly from each other (Table **3**). Pacella, Hruska, Steudte-Schmiedgen, George, & Delahanty [60] and Petrowski *et al.* [55] collected HCC relatively shortly after trauma exposure (*i.e*., injury *vs.* motor vehicle crash). Both reported positive associations, with higher HCC 30 days post-injury predicting bigger increases in PTSD symptom severity 60 days post-injury [60], and higher HCC 10 days post-motor-vehicle-crash predicting higher avoidance behavior, but no other PTSD symptom clusters or overall symptomatology three months later [55]. In contrast to that approach, Steudte-Schmiedgen *et al.* [46] collected hair samples from soldiers before deployment (*i.e*., before potential new-onset trauma exposure) and found lower baseline HCC to predict bigger increases in PTSD symptoms upon new-onset trauma exposure. Lastly, Sopp, Michael, Lass-Hennemann, Haim-Nachum, & Lommen [56] reported no predictive value of baseline HCC for PTSD symptom severity in Dutch firefighters six and 12 months later, without taking into account new-onset trauma exposure. Importantly, this corresponds with the findings from Steudte-Schmiedgen *et al.* [46], who also reported no predictive value when not accounting for additional trauma exposure.

### 3.4 HCC as an Intervention-related Biomarker in Trauma/PTSD

Currently, only two studies have reported on HCC over the course of interventions in the context of trauma/PTSD (Table **4**). Both followed several weeks of intense in-patient trauma-focused therapy, which effectively reduced PTSD symptomatology, albeit without including an untreated control group. Although Woud *et al.* found that a novel cognitive-bias-modification training was beneficial for PTSD symptomatology, this effect was not accompanied by HCC changes [83]. Hummel *et al.* [63] reported increases in HCC from pre-assessment to the five-month follow-up, but not to the post-assessment directly after treatment, potentially indicating the relevance of a longer assessment period. While they did not find a predictive effect of pre-assessment HCC or changes in HCC for PTSD symptom change specifically, lower HCC before treatment was observed to predict less improvement in overall clinical symptomatology from pre- to post-treatment.

### 3.5 Assessment of Reporting Standards and Study Quality

For data on reporting standards and study quality, please see Table **5**.

## 4 DISCUSSION

The current systematic review aimed to update the model by Steudte-Schmiedgen *et al.* [4] on HCC in trauma/PTSD in the context of the framework brought forth by Engel *et al.* [3] as well as with respect to reporting standards/study quality. We identified 31 studies (*n* = 3,576 participants). For proximal trauma exposure or individuals living under high-stress circumstances, we found a more homogeneous picture than for more temporally distal trauma exposure, confirming the predictions of cortisol hypersecretion during/immediately after trauma exposure [4]. For more distant trauma exposure, fewer studies existed and the picture was markedly less clear; therefore, the prediction and exact pattern of hyposecretion with increasing distance to the traumatic event can currently be neither confirmed nor refuted. For prognostic and longitudinal questions, the literature is still sparse, with tentative suggestions of both cortisol hyper- and hyposecretion predicting worse clinical outcomes, depending on the timing of assessment relative to trauma exposure. Similarly, for HCC as an intervention-related biomarker, the few available findings are conflicting. Parts of this heterogeneity may be explained by heterogeneous reporting styles and study quality.

### 4.1 HCC Research in Trauma/PTSD

With regard to HCC as a diagnostic biomarker in the context of trauma/PTSD, a prediction of the model of Steudte-Schmiedgen *et al.* [4] that shows relatively strong empirical evidence is the proposed non-linear timeline of endocrine changes following trauma exposure/PTSD. Among the cross-sectional evidence, all studies focusing on recent or ongoing trauma exposure (threat) reported elevated HCC compared to the respective control groups [39, 40, 43, 48-50 and 42, albeit at trend level]. Tentative support for this observation also emerged from the correlational data, with positive associations of HCC with PTSD symptomatology only in studies focusing on ongoing/recent trauma exposure. This repeated finding of long-term elevated HPA axis activity during or immediately after exposure meshes well with the works of Khoury, Bosquet Enlow, Plamondon, & Lyons-Ruth on adversity [24] also suggesting the timing of exposure to be a central factor for HPA axis secretion patterns.

**Fig. (2):**
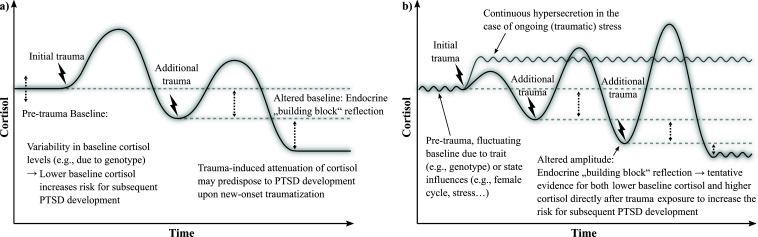
Model **a**) depicts the original integrative model by Steudte-Schmiedgen and colleagues [4] linking long-term cortisol secretion, trauma exposure, and subsequent PTSD development. The model proposes that trauma exposure leads to an initial hypersecretion of cortisol, which, over time, turns into a dose-dependent attenuation of secretion. Thus, cortisol secretion shows an endocrine “building block effect”, matching the clinical observation of higher PTSD risk with multiple trauma exposure. Model **b**) reflects the updated and extended model including the proposed trajectory for ongoing traumatic stress with continuous hypersecretion, as well as an alternative plausible secretion pattern following multiple trauma exposure. Currently, the literature cannot conclusively resolve which trajectory is more accurate: the staircase-shaped one proposed in (**a**), with changes particularly of the baseline secretion, or a sinus-shaped trajectory with changes also in the amplitude, as proposed in (**b**), is more accurate. Methodologically sound longitudinal studies are needed.

Further, the available literature suggests no reversion to hyposecretion when such high-stress conditions last for years or decades [*e.g*., 40, 42]. This is an important psychoneuroendocrine contribution to the clinical discussion about whether ”posttraumatic stress disorder“ is an apt description for such cases or new terminology such as “continuous trauma disorder” [89] is required. Although already implied in the model of Steudte-Schmiedgen *et al.* [4], this finding emerged as so pronounced and different from the assumed two-staged trajectory that we decided to integrate it as a distinct pattern of secretion into an updated version of the model (Fig. **2**).

The picture is remarkably less clear for studies focusing on more distant trauma exposure or not reporting any estimate for timing, with considerably more heterogeneity for group differences or correlational studies. This becomes particularly evident in the study of Heller *et al.* [50]. In their direct contrast of individuals exposed to violence within the past 12 months *versus* those longer than 12 months ago, associations (namely, positive ones with the time since trauma exposure) only emerged in the recently exposed group. This makes it currently impossible to test the assumptions of the model by Steudte-Schmiedgen and colleagues [4] of a staircase-shaped trajectory with decreased baseline secretion, but unchanged cortisol peaks following trauma exposure. Currently, the literature would also support a sinus-shaped trajectory with changes in not only the baseline secretion but also the amplitude of secretion peaks, which we added as a second possible secretion pattern (Fig. **2**).

From a methodological viewpoint, it is not surprising that findings for more distal trauma exposure are more heterogeneous. Bigger differences in individual trajectories (*e.g*., due to exposure to further traumatic or stressful experiences on the one hand, or the experiencing of helpful interventions or social support on the other) may play a role, as well as the increased effects of memory biases with more long-term recollection. These factors may also be relevant in the observation that consistent associations between HCC and clinical data rather emerge from group-level analyses than from correlational ones with symptom severity. This so-called “lack of psychoneuroendocrine covariance” [19] is in part attributed to inherent issues of self-report. In particular, for PTSD, which is increasingly understood as a highly fluctuating condition with great inter- and intraindividual differences in symptom patterns [90], recent insights from smartphone-based ecological momentary assessment confirm the difficulties patients have with aptly reporting the mean severity of their symptoms over longer periods of time [91]. However, studies matching biological markers with such everyday life assessments of symptom severity over a fixed period of time are still pending.

In general, prospective and longitudinal data on HCC in trauma/PTSD are sparse. The four studies available greatly differ with respect to their design, which might explain what initially appear to be heterogeneous results: while Pacella *et al.* [60] and Petrowski *et al.* [55] both reported positive associations, Steudte-Schmiedgen *et al.* [46] found inverse ones, and Sopp *et al.* [56] no associations of HCC with later PTSD symptom severity. However, in accordance with the predictions of the model by Steudte-Schmiedgen and colleagues [4], both increased long-term cortisol secretion in the acute phase of trauma exposure as studied in [55, 60] as well as baseline low secretion in a non-stressful environment [such as might have been the case for soldiers before deployment, 46] may be indicative of higher susceptibility for PTSD symptomatology. As previously stated, recent data from animal studies challenge the notion that the first two studies provide insights into pre-traumatic HPA axis activity, as they both collected HCC after trauma exposure [18]. Further, the similarly-designed studies of Sopp *et al.* [56] on firefighters and Steudte-Schmiedgen *et al.* [46] on soldiers show the importance of integrating a holistic overview of new-onset trauma exposure into predictive analyses, again providing evidence for the proposed “building block effect” of multiple trauma exposure [4]. Importantly, beyond the scope of our manuscript, studies have found higher/lower HCC, depending on the timing of assessment relative to trauma exposure, to predict general psychopathology [55], alcohol consumption [92], or stress exposure in the context of trauma/PTSD [53], supporting the general relevance of HCC as a prospective marker.

With regard to HCC as an intervention-related biomarker in the context of trauma/PTSD, our systematic search only yielded two studies: one reporting HCC increases over the course of the intervention [63], meshing well with the prediction by Steudte-Schmiedgen *et al.* of long-term cortisol attenuation [4], but the other reporting no changes. Thus, further studies - particularly ones incorporating untreated control groups - are needed for an apt interpretation of the results. With regard to pre-treatment HCC as a predictor for therapy success, the findings of Hummel *et al.* [63] with no specific effects for PTSD symptomatology, but lower pre-treatment HCC predicting less overall improvement of clinical symptomatology mesh well with similar results of Fischer *et al.* [93] for depression and anxiety symptomatology. Finally, only two studies could be identified mapping HCC over a longer period of time after trauma exposure in an adult population. Petrowski and colleagues showed an increase in HCC in individuals with any subsequent psychiatric diagnosis three months after trauma exposure [55], but, due to insufficient numbers of individuals having developed PTSD, they did not separately analyze PTSD and non-PTSD groups, or individual HCC trajectories. Steudte-Schmiedgen *et al.* [4] also reported an increase between HCC before and 12 months after deployment, also without specifically focusing on individual trajectories. Thus, there remains a lack of insight into the specific processes of post-traumatic dysregulation.

In summary, the last decade of HCC research in trauma/PTSD has yielded pivotal insights into the neurobiology of PTSD and provided invaluable additions to the more short-term blood, saliva, and urine assessments. Corresponding to the prediction of seminal models [4, 20] and meta-analytic findings from adjunct areas of research [19, 24], the available literature suggests long-term cortisol hypersecretion during or immediately after chronic (traumatic) stress, with less consistent insights available into HPA secretion patterns years or decades after (traumatic) stress. Similar hypersecretion has been repeatedly shown during initial/ early phases of many psychological conditions reviewed in [21]. This, as well as the non-PTSD-specific findings of the predictive effects of pre-treatment HCC for therapy outcome [63, 93] leads to the question of the shared *versus* specific mechanisms of such HPA axis activity patterns for clinical conditions. Notably, it is often impossible to distinguish the effects of psychological conditions on the one hand and chronic stress on the other. For instance, a possible explanation for the HPA axis alteration in PTSD, TE, and other clinical groups, along with the genuine effects of trauma exposure, might be that living conditions associated with a higher risk for trauma exposure typically also entail higher rates of other stressors, and that all clinical conditions constitute chronic stress. At the same time, long-term living with a chronic psychological condition might lead to fatigue or loss of energy, social withdrawal, and difficulties resulting in fewer potential stressors, which might explain the seemingly contrasting observation of Khoury *et al.* of HCC elevations visible only in subclinical - but not clinical - samples with a history of adversity [24]. Again, this highlights the need to carefully report on a holistic picture of lifetime trauma exposure as well as subjectively experienced daily life stress for conclusive insights into the exact mechanisms of the HPA axis.

Contrary to earlier concepts, it has become increasingly clear that HCC, and cortisol alterations per se, may not be specific “universal fix-it-all” [19] biomarkers for singular psychological conditions. Importantly, this is a verdict increasingly prevalent for biomarkers in general [94]. Rather, cortisol alterations seem to be indicative of general stress-related processes, with the picture relatively clear for the first (*i.e*., hypersecretion during/immediately after substantial/ traumatic stress), but considerably heterogeneous for the second of the proposed two-staged process. While the model by Steudte-Schmiedgen *et al.* assumes a roughly staircase-shaped transition upon multiple incidents of trauma exposure, with hypersecretion attenuated but hyposecretion intensified with every new traumatic experience [4], an updated version of the model (Fig. **2**) additionally contains the observed hypersecreting trajectory of individuals remaining under traumatic stress or insecure living conditions. Further, based on the literature, it is currently more plausible that repeated exposure leads to changes of the amplitude (*i.e*., both intensified hyper- and hyposecretion, b), as opposed to the baseline of secretion (*i.e*., intensified hypo-, but attenuated hypersecretion, a), extending on the previously predicted increases only for hyposecretion [4]. However, further - and especially longitudinal - studies are required for a conclusive representation of the biological processes.

### 4.2 Reporting Standards and Study Quality

With regard to the quality of the included studies, the picture emerged as heterogeneous. Notably, no study received an overall rating of “very good” (*M* = 42.8, *SD* = 14.6, range = 12.5-72.2%). Sixteen of the 31 included studies received an overall negative quality rating. Importantly, our data show that methodological rigor actually decreased over the time HCC was implemented in trauma/PTSD research. Although seemingly counterintuitive considering the increased knowledge about potential confounders *e.g*., [14, 19], this may be explained by the fact that HCC has evolved from the research focus of a few highly specialized groups to an interesting, relatively easy-to-implement add-on for multiple clinical studies. It must be emphasized, however, that low ratings of reporting standards do not necessarily mean that a variable had not been accounted for. The ratings can only depict whether a variable and the respective measures taken were reported in the manuscript. In some instances, it is plausible that certain variables were not reported due to them being unproblematic (*e.g*., no reporting of ethnicity when it did not differ between study participants). However, such omissions may lead to subsequent research neglecting central information as they follow the example of previous studies. Unsurprisingly, the highest ratings emerged for trait participant characteristics (*M* = 70.0, *SD* = 17.9%), in all likelihood caused by the overlap with general reporting standards of empirical studies (*e.g*. age, sex). In contrast, the lowest levels were achieved for participants’ health-related characteristics (*M* = 32.9, *SD* = 24.1%) and substance and medication intake (*M* = 34.3, *SD* = 25.8%). This, again, is not surprising considering the often complex picture of comorbidities and medical treatments in clinical samples. However, these two factors have a strong potential of systematically influencing results [with particular relevance to the intake of glucocorticoid-containing medication, see, *e.g*., 95], which highlights the necessity of striving for high standards of reporting.

With regard to the reporting standards of trauma/PTSD-associated characteristics, 15 of the 31 studies implemented clinical interviews to assess PTSD status and symptom severity, 14 used self-report instruments, and three did not report PTSD symptomatology at all. On LTE, studies reported in a very heterogeneous fashion, with a holistic overview of the type, number, frequency, and timing of LTE being rare. Most prominently, among the 31 studies, only two assessed the exact timing of previous trauma exposure. Thirteen reported timing for certain parts of LTE, such as the focus trauma, while 15 did not report the timing of LTE at all.

### 4.3 Strengths and Limitations

A limitation and, at the same time, the strength of the study is the focus on lifetime trauma exposure, following the criteria for trauma exposure proposed by the DSM-IV/DSM-5 trauma criteria [1, 27]. Although this is considered the current gold standard and provides a necessary extension of the works of Khoury *et al.* and Stalder *et al.* on relatively broadly defined adversity [24] and stress [19], it often led to complex inclusion decisions. Frequently, experiences meeting and not meeting the DSM-IV/DSM-5 criteria for traumatic events were assessed in an entangled fashion summarized under “adversity”, which then led to an exclusion from our systematic review. Further, as only published and peer-reviewed studies were included, an influence of publication bias is plausible. Moreover, the included studies were considerably heterogeneous, particularly with regard to reporting standards. Importantly, there often is a lack of consensus on which covariates to be considered as relevant confounders. Thus, we decided to rate in a relatively liberal fashion, and to acknowledge any attempt to control for confounders (*e.g*., both the broad exclusion of any comorbidity, as well as the focus on specific psychological comorbidities led to a favorable rating). This, in some cases, is certainly associated with a loss of specificity in the quality ratings. However, considering the plethora of potential confounders and their potential interactions, a more precise rating is not feasible until more is known about the underlying mechanisms.

### 4.4 Outlook and Recommendations

Although evidence is accumulating that cortisol should be interpreted as a transdiagnostic instead of a specific biomarker for trauma/PTSD, this does not dispute the fact that the HPA axis is crucially involved in the processing of (posttraumatic) stress. Along with being a potential sequela of trauma exposure, its dysregulations have also been discussed as a risk factor for the development of PTSD [4], and first interesting insights have suggested its potential for interventions [96, 97]. Thus, research on the complex patterns of the HPA axis and its interacting systems can still be considered highly relevant for a better understanding of the biopsychosocial processes underlying trauma and PTSD. Particularly important open questions are a) the long-term consequences of different types of trauma exposure (*e.g*., singular *vs.* intermittent *vs*. chronic exposure, childhood *vs*. adulthood exposure) or PTSD for the HPA axis; b) the exact secretory processes underlying hyper- and hyposecretion, (*e.g*., a general change of basal secretion *vs*. a change in reactivity towards stressors/trauma-related triggers *vs*. a change of recovery after such stressors/triggers); c) the exact time point(s) of reversion from hyper- to hyposecretion; d) the individual and situational factors influencing the extent and time point of dysregulation and reversion; e) the potential psychological counterparts of hyper- *versus* hyposecretion; and f) the ramifications of such alterations for interacting neurobiological systems (*e.g*., noradrenergic pathways, inflammation, epigenetic processes, *etc*.). To answer these questions, well-powered and particularly longitudinal studies repeatedly (ideally every few weeks/months) assessing HCC following trauma exposure with high methodological rigor, combining different ways of cortisol (and related biomarker) assessment, and utilizing state-of-the-art advances in psychoendocrine research are needed.

As a first point, recent years have brought forth, methodological studies on factors influencing HCC 18 on the time scale of hormonal incorporation in hair, 98 on the role of sweat for HCC, or 99 on the comparability of international laboratory results, to only name a few. In this tradition, future studies are needed that give a precise estimate about which variables to exclude, to control for statistically, or to ignore with a clear conscience. Further, the research field of psychoneuroendocrinology will benefit from important technical advances. In recent years, it has become feasible to assess cortisol secretion in a continuous, long-term, and non-to-minimally-invasive fashion *via* wearable solutions. Examples are the real-time assessment of cortisol from tear fluid *via* contact-lens-based sensors [98-100], or from sweat using patch sensors *e.g*., [101-103] or wrist watches [104]. Following approaches from adjoining fields the monitoring of food consumption, [105], tooth-mounted sensors for a real-time assessment of salivary cortisol are also conceivable. One central advantage of such techniques compared to HCC analyses is that they may provide insights into exact timelines of secretion patterns, which, when paired with ecological momentary assessment of symptomatology, will be an invaluable asset for fluctuating conditions such as PTSD. A further, crucial development is the application of complex statistical procedures for psychoneuroendocrine questions. Although the general consensus has been to focus on longitudinal studies with multiple time points of hormonal assessment as well as on secretion patterns of different, interacting agents, such endeavors are complex and require statistical analyses exceeding the traditional, general-linear-model-based approaches. Even though hormonal ratios (*e.g*., the cortisol:DHEA or cortisol:cortisone ratio) are often utilized for an easier handling of multiple biomarkers, those are associated with inherent issues such as their asymmetry requiring non-parametric analysis methods, the complexity of interpretation, and inherent loss of information [106]. Thus, statistical methods combining multivariate and often highly collinear data in a recursive, data-driven search for an optimal model are an exciting new way of integrating complex information. Although there are first applications in the context of endocrine markers for trauma/PTSD [61, 107], the broad application in the context of HCC analyses is still pending. Finally, shared research guidelines advanced by the field are urgently needed. Thus, we would like to end this systematic review with a series of (crucial, but non-exhaustive) recommendations for researchers studying HCC in the context of trauma/PTSD. Some of the points are relevant for trauma/PTSD or HCC research in general. However, in the interplay of studying both, many aspects warrant extra care and may play a role in the frequently observed “lack of psychoendocrine covariance” [19].

Factors relevant for the *assessment of trauma/PTSD* in general, but particularly for HCC research:

- As a first and central point, we recommend meticulously defining each researched concept upon introduction. Although “trauma exposure” is clearly defined in the DSM-5, for the partly overlapping, partly related concept of adversity (particularly during childhood), clear consensus definitions have yet to be established [*e.g*., 108]. For events fulfilling DSM-5 criteria, we suggest using and explaining the terms “LTE” (*i.e*., at any point of the subjects’ lives); “adulthood trauma exposure” (*i.e*., after adolescence); and “childhood trauma exposure” (*i.e*., before or during adolescence). Similarly, related constructs such as “child maltreatment” (as defined by the Center of Disease Control and Prevention, 109: emotional, physical, and sexual abuse, emotional and physical neglect) or “adverse childhood events” (additionally encompassing unstable living conditions due to parental separation, imprisonment, or mental health problems see, *e.g*., [110]) should be clearly defined.
- In general, we recommend reporting as holistically as possible on LTE. Particularly relevant characteristics are type, frequency, duration, and timing of any experienced trauma exposure (with regard to the amount of time between exposure and assessment, as well as the individual developmental phase of exposure). At the minimum, researchers should report on the initial, the most recent, the most severe, and the focus trauma of the study and, ideally, integrate objective information on LTE. Until this becomes the standard, it will neither be possible to clear up the heterogeneous results, particularly with respect to the postulated long-term hyposecretion, nor to study the psychoneuroendocrine consequences of different trauma characteristics such as type, duration, or frequency of trauma exposure.
- Based on the important findings on the relevance of the ongoing threat of (traumatic) stress, we recommend asking for the current perceived threat of trauma as well as the daily life stress experienced. It may also be fruitful to assess previous non-traumatic adversity to gain insights into shared and differing mechanisms. Although tedious and effortful, only such a diligent procedure will allow the testing of the building block model and a conclusive interpretation of the seemingly conflicting results of hyper-, hypo-, or unchanged cortisol secretion in trauma/PTSD.
- Importantly, such a holistic picture is very difficult to achieve using trauma or life event checklists. Although such a procedure is preferable to assumptions about the presence of traumatic events from factors such as the place of living (for instance, in areas where acts of war or natural disasters have taken place), such checklists have several shortcomings. Firstly, our clinical impression is that they tend to evoke over-reporting of events, as it is almost impossible to pose the questions in an unambiguous, yet universally valid fashion. Secondly, they typically cannot depict complex exposure situations with intermittent or chronic exposure. Lastly, such questionnaires often put considerable strain on participants, and particularly on those with a PTSD diagnosis. In clinical interviews on trauma history such as the Clinically-Administered PTSD Scale for DSM-5 [CAPS-5, 67], further inquiries can be posed, answers can be rated within the individual context, and participants can be aptly guided by (trained) interviewers. Thus, we advise going the extra mile of applying clinical interviews in favor of more robust results.
- Lastly, we suggest taking extra care with regard to the control group. Especially in the case of PTSD *versus* TE, there are sometimes only small differences in symptom severity resulting in the endorsement or rejection of a PTSD diagnosis. Here, studies explicitly contrasting individuals without PTSD and no, low, medium, or relatively high symptom severity is warranted.

Factors relevant for the *assessment of HCC* in general, but particularly for trauma/PTSD research:

- As the most important point here, we strongly recommend to report trait confounders, substance and medication intake, health-related variables, hair-related variables, and sampling and analysis factors in as holistic a fashion as possible. For lack of published guidelines on HCC confounders, we propose following the seminal CoAL guidelines [37, notwithstanding their differing focus on blood, urine, and saliva sampling] and the reporting standards examined in this systematic review. Although the influence of some of the possible confounders is not (yet) clarified, this makes it even more relevant to report and, if the sample size allows, analyse respective data.
- Along the same vein, clear linguistics is important. Although it is plausible to use general descriptions (*e.g*., report the exclusion of “severe illness” or “medication influencing the endocrine system” for reasons of brevity), imprecise terminology leads to heterogeneous methodology and thus lack of comparability between studies.
- We recommend not to compare absolute HCC values/ differences across different studies/laboratories/assess-ment methods, as they might vary substantially with the implemented method. For example, it has repeatedly been shown that immunoassays tend to yield higher HCC than LC-MS/ MS methods [for a summary, see 19, *e.g*., 99]. Of course, the comparison of relative results is necessary and typically feasible for studies with slightly differing methodologies.
- We strongly suggest practicing open and reproducible science in psychoneuroendocrine research [for recommendations, see 111]. From smaller steps like choosing box-, bean-, or violin plots instead of traditional bar charts to better illustrate the variance of the data, to soundly reporting and imputing non-detectable and outlying values [38], to sharing whole data sets with the community, every measure taken improves the interpretability and replicability of findings. Further, such practices also foster secondary analyses of potential confounders.

## CONCLUSION

To conclude, the last decade of HCC research in trauma/PTSD has seen central findings integrated into seminal models, considerable heterogeneity, and many lessons learned. With continuing progress in the next decade, the fundament of the available pioneer work, the gained methodological knowledge, and the technical advances, it is reasonable to hope for further breakthroughs regarding psychoneuroendocrine mechanisms underlying trauma/PTSD.

## Figures and Tables

**Fig. (1):**
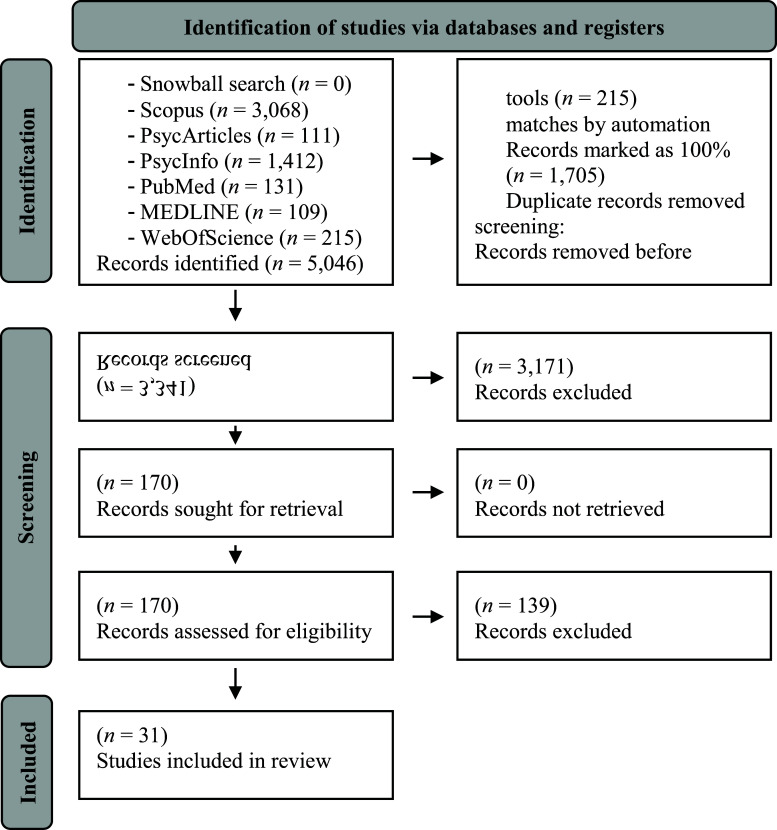
PRISMA flowchart illustrating the process of study selection.

**Table 1:** Overview of studies on HCC as a diagnostic biomarker in trauma/PTSD: Group differences.

**Author (Year)**	**Assessment Times**	**Sample**	**Sample for HCC Analyses**	**Brief Sample Description, ** **Country**	**Age ** **(*M, SD*)**	**Sex (% Female)**	**BMI (*M, SD*)**	**Type of Focus Trauma**	**Assessment of PTSD**	**Assessment of LTE**	**Timing of LTE**	**Hair Segments, Analysis Method**	**Results with Respect to HCC Group Differences**
Steudte *et al.*, 2011 [39]	Singular	PTSD (*n* = 10)	PTSD (*n* = 10)	Young adults, mixed gender (Uganda)	19.2 (3.2)	60	22.42 (1.90)	civil war	Screening: PDS > 11; CAPS	Yes, types (but not frequency) of exposure *via* self-developed LTE checklist	n.r., but 60% of PTSD and 22.2% of TE experienced trauma within the last year	≤ 3 cm, CLIA	PTSD > TE
		TE (*n* = 22)	TE (*n* = 17)		20.1 (5.7)	35.3	21.44 (2.24)		Screening: PDS = 0; CAPS				
Steudte *et al.*, 2013 [45]	Singular	PTSD (*n* = 28)	PTSD (*n* = 25)	Adults, mixed age/gender (Germany)	36.84 (11.25)	96	23.90 (3.12)	mixed, mostly civilian	DIA-X/ M-CIDI, PDS	Yes, THQ	THQ (3-6 m = 4.3 *vs*. 0%, 6-36 m = 13.0 *vs*. 8%, 36-60 m = 8.7 *vs*. 16%, > 60 m = 73.9 *vs*. 76%)	2 x 3 cm, LC-MS/ MS	PTSD = TE < NTE
		TE (*n* = 27)	TE (*n* = 25)		41.72 (12.32	92	23.77 (3.91)						
		NTE (*n* = 32)	NTE (*n* = 28)		37.61 (14.05)	89.3	23.40 (3.05)						
Gao *et al.*, 2014 [43]	Singular	TE (*n* = 20)	TE (*n* = 20)	Adults, mixed age/ gender (China)	45.0 (14.2)	40	n.r.	Earthquake	CAPS ≤ 39	n.r.	Only for focus trauma: ≤ 6w	1 x 1.5 cm, LC-MS/ MS	TE > NTE
		NTE (*n* = 23)	NTE (*n* = 23)		41.5 (12.8)	39.1							
Steudte-Schmiedgen *et al.*, 2015 [46]	t0 (before deployment), t1 (12m after deployment)	TE/PTSD (*n* = 113)	TE (*n* = 113)	Young, male soldiers (Germany)	27.68 (6.11)	0	25.45 (2.69)	Combat & civilian	DIA-X/ M-CIDI; PCL-C	Yes, DIA-X/ M-CIDI	n.r.	2 cm, LC-MS/MS	Baseline: TE/PTSD < NTE (non-significant trend)
		NTE (*n* = 129)	NTE (*n* = 129)										
Groër *et al.*, 2016 [52]	Singular	CSA+ (*n* = 27)	CSA+ (*n* = 27)	Female veterans, mixed age (U.S.)	47.3 (10.7)	100	28.7 (5.1)	Focus on sexual assault	PCL-M	Only childhood/ civilian/ military sexual assaults (rape/ attempted rape) with self- developed questionnaire	n.r.	3 cm, ELISA	CSA+ < CSA-
		CSA- (*n* = 54)	CSA- (*n* = 54)		45.6 (10.5)	100	29.7 (6.8)						
Boeckel *et al.*, 2017 [48]	Singular	IPV+ (*n* = 27)	IPV+ (*n* = 27)	Female adults, mixed age (Brazil)	34.15 (6.52)	100	n.r.	IPV	PSS-SR	n.r.	Only for IPV: ≤ 1y	1 cm, ELISA	IPV+ > IPV-
		IPV- (*n* = 25)	IPV- (*n* = 25)		36.03 (8.31)	100							
Mewes *et al.*, 2017 [49]	Singular	Asylum seekers: PTSD+ (*n* = 32)	Asylum seekers: PTSD+ (*n* = 32)	Adults, mixed age/gender (Germany)	32.8 (6.9)	56	26.6 (5.1)	mixed	PDS, SCID-I PTSD section	PDS extended by HTQ items on traumatic events often experienced by refugees	n.r.	2 cm, CLIA	Asylum seekers: PTSD+ = Asylum seekers: PTSD- > immigrants (with non-immigrants in between)
		Asylum seekers: PTSD- (*n* = 24)	Asylum seekers: PTSD- (*n* = 24)		32.0 (7.6)	42	24.0 (3.2)						
		Immigrants (*n* = 24)	Immigrants (*n* = 24)		24.3 (2.7)	0	26.2 (3.5)						
		Non-immigrants (*n* = 28)	Non-immigrants (*n* = 28)		25.9 (3.8)	0	22.8 (2.2)						
Morris *et al.*, 2017 [53]	t0, t1 (t0 + 3m)	CA+ violence (*n* = 12)	CA+violence (*n* = 12)	Young, female adults (U.S.)	23.6 (3.6)	100	n.r.	IPV, CA	SCID-I, CAPS	n.r.	Only for violence: ≤ 3m	3 cm, CLIA	No group differences at baseline
		CA-violence (*n* = 7)	CA-violence (*n* = 7)		22.6 (2.3)	100							
		NTE (*n* = 15)	NTE (*n* = 15)		25.7 (3.4)	100							
Heller *et al.*, 2018 [50]	Singular	Violence- (*n* = 37)	Violence- (*n* = 22)	Female sex workers, mixed age (Kenya)	32.1 (n.r.)	100	n.r.	Physical, emotional, sexual (gender-based) violence	PCL-C	Yes, for physical, emotional, and sexual violence (categorical: ≤ *vs*. > 12m)	Only for physical, emotional, sexual violence (categorical: ≤ *vs*. > 12m)	3 cm, ELISA	Violence+ recently > violence- = Violence+ remotely
		Violence+ remotely (*n* = 134)	Violence+ remotely (*n* = 71)		35.6 (n.r.)	100							
		Violence+ recently (*n* = 112)	Violence+ recently (*n* = 48)		31.0 (n.r.)	100							
van Zuiden *et al.*, 2019 [44]	Singular	PTSD (*n* = 14)	PTSD (*n* = 13)	Female police officers, mixed age (The Netherlands)	42 (7.96)	100	25 (4.14)	Focus police-related trauma	CAPS ≥ 45	In police context: PLES	n.r.	3 cm, ELISA	PTSD < TE
		TE (*n* = 16)	TE (*n* = 15)		38 (9.98)	100	26.43 (3.26)		CAPS ≤ 15				
Castro-Vale *et al.*, 2020 [47]	Singular	PTSD (*n* = 31)	PTSD (*n* = 31)	Male elderly veterans (Portugal)	64.7 (3.5)	0	28.2 (3.6)	Focus on war trauma	CAPS ≥ 50, frequency ≥ 1, intensity ≥ 2	yes, CAPS, adapted WEQ	only for focus trauma war: 40y	1-3 cm, LC-MS/MS	No group differences
		TE (*n* = 28)	TE (*n* = 28)		65.8 (3.3)	0	27.2 (2.4)		CAPS < 50, frequency < 1, intensity < 2				
van den Heuvel *el al*., 2020 [40]	Singular	PTSD (*n* = 307)	PTSD (*n* = 110)	Female adults, mixed age (South Africa)	40.8 (11.4)	100	n.r.	mixed	CAPS-5 ≥ 23	yes, LEC-5	Only for subjectively most severe traumatic event	3 cm, LC-MS/MS	PTSD > TE
		TE (*n* = 321)	TE (*n* = 106)		46.9 (14.4)	100			CAPS-5 ≤ 22				
Lynch *et al.*, 2022 [51]	Singular	Women from the general population (*n* = 689)	Women from the general population (*n* = 470)	Female adults, mixed age (Iceland)	52.9 (11.0)	100	27.6 (5.5)	Violence	PC-PTSD	Yes, LSC-R	Yes, LSC-R	3 cm, LC-MS/ MS	Exposed to violence > non-exposed to violence
Schumacher *et al.*, 2022 [41]	Singular	PTSD (*n* = 19)	PTSD (*n* = 19)	Male adults, mixed age (Germany)	38.53 (10.23)	0	26.50 (3.61)	Deployment-related trauma	CAPS	n.r.	Only for subjectively most severe traumatic event: PTSD: 9.53 (6.79), TE: 8.89 (7.05)	1.5 cm, CLIA	PTSD = TC > NTC
		TE (*n* = 10)	TE (*n* = 10)		40.90 (9.47)	0	25.43 (1.90)						
		NTE (*n* = 10)	NTE (*n* = 10)		27.60 (4.03)	0	25.30 (1.97)						
Yirmiya *et al.*, 2022 [42]	Singular	78 samples (not specified how many from TE/PTSD or NTE)	78 samples (not specified how many from TE/ PTSD or NTE)	Female adults, mixed age (Israel)	ca. 38 (n.r.)	100	n.r.	Mixed	PCL-5	n.r.	n.r.	3 cm, ELISA	TE/PTSD > NTE (trend level)

**Notes:** CA = childhood abuse; CAPS = Clinically-Administered PTSD Scale [66]; CAPS-5 = Clinically-Administered PTSD Scale for DSM-5 [67]; CLIA = chemiluminescence immunoassay; CSA = childhood sexual abuse; DIA-X/M-CIDI = Diagnostisches Expertensystem für Psychische Störungen [68]/Composite International Diagnostic Interview – Military [69]; ELISA = Enzyme-linked immunosorbent assay; HCC = hair cortisol concentration; HTQ = Harvard Trauma Questionnaire [70]; IPV = intimate partner violence; LC-MS/MS = liquid chromatography tandem mass spectrometry; LEC-5 = Life Event Checklist for DSM-5 [71]; LSC-R = Life Stressor Checklist – Revised [72]; LTE = lifetime trauma exposure; M = mean; m = months; n = number of participants; n.r. = not reported; NTE = non-trauma-exposed participants; PCL-5 = PTSD Checklist for DSM-5 [73]; PCL-C = PTSD Checklist – Civilian Version [74]; PCL-M = PTSD Checklist – Military Version [75]; PC-PTSD = Primary Care PTSD Screen [76]; PDS = Posttraumatic Diagnostic Scale [77]; PLES = Police Life Events Checklist [78]; PSS-SR = PTSD Symptom Scale – Self Report [79]; PTSD = posttraumatic stress disorder; SCID-I = Structured Clinical Interview for DSM-IV [80]; SD = standard deviation; TE = trauma-exposed participants who did not fulfil the criteria for a PTSD diagnosis; THQ = Trauma History Questionnaire [81]; w = weeks; WEQ = War-Exposure Questionnaire [82].

**Table 2:** Overview of studies on HCC as a diagnostic biomarker in trauma/PTSD: Associations with characteristics of PTSD symptomatology/LTE.

**Author (Year)**	**Assessment times**	**Sample**	**Sample for HCC Analyses**	**Brief Sample Description, Country**	**Age (*M, SD*)**	**Sex (% Female)**	**BMI (*M, SD*)**	**Type of Focus Trauma**	**Assessment of PTSD**	**Assessment of LTE**	**Timing of LTE**	**Hair Segments, Analysis Method**	**Results with Respect to HCC Associations**
Steudte *et al.*, 2011 [39]	Singular	PTSD (*n* = 10)	PTSD (*n* = 10)	Young adults, mixed gender (Uganda)	19.2 (3.2)	60	22.42 (1.90)	Civil war	Screening: PDS > 11; CAPS	Yes, types (but not frequency) of exposure *via* a self-developed LTE checklist	n.r., but 60% of PTSD and 22.2% of TE experien-ced trauma within the last year	≤ 3 cm, CLIA	Positive association with a number of traumatic events
		TE (*n* = 22)	TE (*n* = 17)		20.1 (5.7)	35.3	21.44 (2.24)		Screening: PDS = 0; CAPS				
Andersen *et al.*, 2013 [59]	Singular	Students confronted with peer death (*n* = 28)	Students confronted with peer death (*n* = 24)	Young adults, mixed gender (U.S.)	20.25 (1.19)	70.8	n.r.	Interpersonal trauma, berea-vement	n.r.	Yes, prior interpersonal trauma/ bereavement	Peer death ≤ 12m	3 cm, CLIA	No association with interpersonal trauma, but an inverse one of prior bereavement experience when experiencing peer death
Steudte *et al.*, 2013 [45]	Singular	PTSD (*n* = 28)	PTSD (*n* = 25)	Adults, mixed age/ gender (Germany)	36.84 (11.25)	96	23.90 (3.12)	Mixed, mostly civilian	DIA-X/M-CIDI, PDS	Yes, THQ	THQ (3-6m = 4.3 *vs*. 0%, 6m - 3y = 13.0 *vs*. 8%, 3 - 5y = 8.7 *vs*. 16%, > 5 y = 73.9 *vs*. 76%)	2 x 3 cm, LC-MS/ MS	Inverse association with severity of intrusions, number and frequency of and time since trauma exposure, but not with overall PTSD symptom severity, avoidance, hyperarousal
		TE (*n* = 27)	TE (*n* = 25)		41.72 (12.32	92	23.77 (3.91)						
		NTE (*n* = 32)	NTE (*n* = 28)		37.61 (14.05)	89.3	23.40 (3.05)						
Steudte-Schmiedgen *et al.*, 2015 [46]	t0 (before deployment), t1 (12 m after deployment)	TE/PTSD (*n* = 113)	TE/PTSD (*n* = 113)	Young, male soldiers (Germany)	27.68 (6.11)	0	25.45 (2.69)	Combat & civilian	DIA-X/M-CIDI; PCL-C	Yes, DIA-X/ M-CIDI	n.r. for baseline LTE	2 cm, LC-MS/ MS	Inverse associations with number of different lifetime stressful, but not traumatic events
		NTE (*n* = 129)	NTE (*n* = 129)										
Bluemke *et al.*, 2017 [31]	Singular	Abducted by LRA+ (*n* = 29)	Abducted by LRA+ (*n* = 29)	Young, male adults (two minors) (Uganda)	21.31 (2.48)	0	n.r.	Abduction by LRA	PDS	Yes, types (but not number) of exposure *via* a self-developed checklist	Only for abduction: M = 8.74, SD = 4,14y before data collection, range = 1 – 17y	≤ 3 cm, CLIA	No associations with traumatic event types experienced or PTSD symptom severity
		Abducted by LRA- (*n* = 35)	Abducted by LRA- (*n* = 35)		21.54 (2.51)	0							
Boeckel *et al.*, 2017 [48]	Singular	IPV+ (*n* = 27)	IPV+ (*n* = 27)	Female adults, mixed age (Brazil)	34.15 (6.52)	100	n.r.	IPV	PSS-SR	n.r.	Only for IPV: ≤ 1y	1 cm, ELISA	No associations with PTSD symptom severity
		IPV- (*n* = 25)	IPV- (*n* = 25)		36.03 (8.31)	100							
Fischer *et al.*, 2017 [65]	Singular	Community-based adults (*n* = 144)	Community-based adults (*n* = 139)	Adults, mixed age/ gender (U.K.)	50.6 (14.6)	72	27.5 (6.0)	mixed	n.r.	Yes, self-developed LTE checklist (categorical: ≤ *vs*. > 12 m)	Yes, self-developed LTE checklist (categorical: ≤ *vs*. > 12 m)	3 cm, CLIA	No associations with LTE and 12 m-TE (yes/no) Positive associations with war experiences, inverse ones with physical neglect and crime victimization (the latter at trend level)
Pacella *et al.*, 2017 [60]	t0 (30d post-injury), t1 (60d post-injury)	TE (*n* = 34)	TE (*n* = 30)	Adults, mixed age/gender (U.S.)	33.1 (12.9)	71	n.r.	Physical injury following accidents or assault	PDS, PCL-C	Yes, PDS	Only for injury (30 /60d)	3 cm, LC-MS/ MS	No associations with symptoms or number of LTE
Heller *et al.*, 2018 [50]	Singular	Violence- (*n* = 37)	Violence- (*n* = 22)	Female sex workers, mixed age (Kenya)	32.1 (n.r.)	100	n.r.	Physical, emotional, sexual (gender-based) violence	PCL-C	Yes, for physical, emotional, sexual violence (categorical: ≤ *vs*. > 12m)	Only for physical, emotional, sexual violence (categorical: ≤ *vs*. > 12m)	3 cm, ELISA	Positive association with time since exposure (only in the last 3.8m), but none with PTSD symptom severity
		Violence+ remotely (*n* = 134)	Violence+ remotely (*n* = 71)		35.6 (n.r.)	100							
		Violence+ recently (*n* = 112)	Violence+ recently (*n* = 48)		31.0 (n.r.)	100							
Schalinski *et al.*, 2019 [61]	Singular	Inpatients, mixed diagnoses (*n* = 183)	Inpatients, mixed diagnoses (*n* = 183)	Adults, mixed age/ gender (Germany)	25.9 (6.7)	44.8	24.9 (5.3)	mixed, focus CA	PSS-I	Yes, LEC	n.r.	3 cm, CLIA	No association with LEC trauma load
		TE/NTE (*n* = 75)	TE/NTE (*n* = 75)		25.4 (6.7)	45.3	23.4 (3.6)						
Söder *et al.*, 2019 [58]	Singular	Psychosis: Clinical (*n* = 43)	Psychosis: Clinical (*n* = 42)	Adults, mixed age/ gender (Germany)	26.2 (8.2)	65.1	22.6 (3.3)	n.r.	SCID-I	Yes, THQ	n.r.	3 cm, CLIA	Positive associations with LTE and CA
		Familial (*n* = 32)	Familial (*n* = 32)		33.3 (12.4)	65.6	24.0 (3.2)						
		Low risk (*n* =35)	Low risk (*n* = 35)		27.3 (9.6)	62.9	21.8 (2.8)						
van Zuiden *et al.*, 2019 [44]	Singular	PTSD (*n* = 14)	PTSD (*n* = 13)	Female police officers, mixed age (The Netherlands)	42 (7.96)	100	25 (4.14)	Focus on police-related trauma	CAPS ≥ 45	In police context: PLES	n.r.	3 cm, ELISA	No association with PTSD symptom severity in PTSD group
		TE (*n* = 16)	TE (*n* = 15)		38 (9.98)	100	26.43 (3.26)		CAPS ≤ 15				
Behnke *et al.*, 2020 [62]	singular	Emergency medical service personnel (*n* = 115)	Emergency medical service personnel (*n* = 53)	Adults, mixed age/ gender (Germany)	Med = 25 (IQR = 14)	43.5	Med = 25.95 (IQR = 6.30)	Mixed, focus on work-related trauma	PCL-5, RESQ-CE	yes, LEC-5	n.r.	1 cm, HR-MS/ MS	No associations with LTE, RESQ-CE, or with PTSD symptom severity
Buchmüller *et al.*, 2020 [54]	Singular	Syrian refugees in a refugee camp in Iraq (*n* = 14)	Syrian refugees in a refugee camp in Iraq (*n* = 14)	Female adults, mixed age (Germany)	34.34 (11.71	100	n.r.	Mixed, focus on refugee adversity	HTQ	Yes, for refugee adversity: self-developed scale	n.r.	2x 3 cm, LC-MS/ MS	In Syrian refugees in camp in Iraq: Positive associations with PTSD symptom severity
		Syrian refugees arrived in Germany on average 2y ago (*n* = 37)	Syrian refugees arrived in Germany on average 2y ago (*n* = 37)		30.04 (5.25)	100							
		Kurdish immigrants/ asylum seekers >10y in Germany (*n* = 38)	Kurdish immigrants/asylum seekers > 10y in Germany (*n* = 38)		34.63 (9.39)	100							
Castro-Vale *et al.*, 2020 [47]	Singular	PTSD (*n* = 31)	PTSD (*n* = 31)	Male elderly veterans (Portugal)	64.7 (3.5)	0	28.2 (3.6)	Focus on war trauma	CAPS ≥ 50, frequency ≥ 1, inten-sity ≥ 2	Yes, CAPS, adapted WEQ	Only for focus trauma war (40y)	1-3 cm, LC-MS/ MS	No associations with war exposure In veterans without lifetime MDD, positive associations with war exposure
		TE (*n* = 28)	TE (*n* = 28)		65.8 (3.3)	0	27.2 (2.4)		CAPS < 50, frequency < 1, intensity < 2				
Petrowski *et al.*, 2020 [55]	t0 (within first 10d after motor vehicle crash), t1 (t0 + 3m)	Motor vehicle crash victims (*n* = 62)	Motor vehicle crash victims (*n* = 61)	Adults, mixed age/ gender (Germany)	43.75 (13.51)	92.9	26.31 (5.46)	Motor vehicle crash	SCID-I, PDS, IES-R	Yes, exclusion of previous trauma *via* THQ, SCID-I	Only for motor vehicle crash	3 cm, LC-MS/ MS	t0 HCC not associated with initial PTSD symptom severity t1 HCC positively associated with initial PTSD symptom severity, particularly hyperarousal
van den Heuvel *el al*., 2020 [40]	Singular	PTSD (*n* = 307)	PTSD (*n* = 110)	Female adults, mixed age (South Africa)	40.8 (11.4)	100	n.r.	Mixed	CAPS-5 ≥ 23	Yes, LEC-5	Only for subjective-ly most severe traumatic event	3 cm, LC-MS/ MS	Positive associations with PTSD symptom severity (in particular with regard to intrusions, changes in cognition/ mood, and arousal) in unadjusted and adjusted models. Positive associations with number of trauma types only in unadjusted models
		TE (*n* = 321)	TE (*n* = 106)		46.9 (14.4)	100			CAPS-5 ≤ 22				
Hummel *et al.*, 2021 [63]	t0 (pre-treatment), t1 (t0 + n.r., duration of treatment: *M* = 63.6, *SD* = 14.5, post-treatment), t2 (t1 + 5m, follow-up)	PTSD (*n* = 52)	PTSD (t0: *n* = 52, t1: *n* = 42, t2: *n* = 27)	Female adults, mixed age (Germany)	41.60 (10.54)	100	27.31 (5.38)	Mixed	SCID-I, diagnostic interview with psychologists, PDS	Yes, PDS checklist	n.r.	3 cm, LC-MS/ MS	No associations with PTSD symptom severity or number of traumatic events
Sopp *et al.*, 2021 [56]	t0, t1 (t0 + 6m), t2 (t0 + 12m)	Firefighters (*n* = 529)	Firefighters (*n* = 371)	Adults, mixed age/ gender (The Netherlands)	38.78 (10.10)	7.28	n.r.	Mixed, focus on work-related trauma	PCL-5	Yes, self-developed questionnaire on work-related trauma, LEC-5	n.r.	2 cm, n.r.	Positive associations with baseline PTSD symptom severity in individuals with average and above-average, but not below-average work-related trauma severity
Spikman *et al.*, 2021 [33]	Singular	Patients with mild traumatic brain injury (*n* = 46)	Patients with mild traumatic brain injury (*n* = 43)	Adults, mixed age/gender (The Netherlands)	38.8 (16.5)	39.5	n.r.	n.r.	IES-R	Not reported	Not reported	2x 1 cm, LC-MS/ MS	No associations with PTSD symptomatology
		Healthy control participants (*n* = 11)	Healthy control participants (*n* = 11)		36.7 (14.2)	36.4							
Woud *et al.*, 2021 [83]	t0 (pre-training), t1 (t0 + 6w, post-training), t2 (t1 + 3m, follow-up)	CBM-APP (*n* = 39)	CBM-APP (t0: *n* = 32, t1: *n* = 30, t2: *n* = 23)	Adults, mixed age/ gender (Germany)	42.41 (12.42)	92.3	n.r.	mixed	CAPS-5, PTCI, PCL-5	Yes, CAPS-5/ LEC-5	n.r.	3 cm, LC-MS/ MS	No associations with PTSD symptom severity and posttraumatic cognition
		Control (*n* = 41)	Control (t0: *n* = 25, t1: *n* = 26, t2: *n* = 19)		39.05 (12.45)	82.9							
Basso *et al.*, 2022 [64]	Singular	Chronic tinnitus patients (*n* = 94)	Chronic tinnitus patients (*n* = 91)	Adults, mixed age/gender (Germany)	51.5 (12.0)	65.9	25.8 (4.6)	mixed	n.r.	Yes, PDS event list	n.r.	1 cm, ELISA	No association with a number of experienced traumatic events
Bob *et al.*, 2022 [57]	Singular	Patients with an initial episode of psychosis (*n* = 56)	Patients with an initial episode of psychosis (*n* = 56)	Female adults, mixed age	28.43 (5.32)	100	n.r.	n.r.	TSC-40	n.r.	n.r.	2 x 1 cm, ELISA	Inverse associations with PTSD symptom severity
Lynch *et al.*, 2022 [51]	Singular	Women from the general population (*n* = 689)	Women from the general population (*n* = 470)	Female adults, mixed age (Iceland)	52.9 (11.0)	100	27.6 (5.5)	violence	PC-PTSD	Yes, LSC-R	Yes, LSC-R	3 cm, LC-MS/ MS	No associations with time since last exposure or age at exposure
Marcil *et al.*, 2022 [32]	Singular	Healthcare workers (*n* = 467)	Healthcare workers (*n* = 372 with 3 cm, *n* = 358 with 6 cm hair sample)	Adults, mixed age/gender (Canada)	40.3 (9.1)	92.4	26.8 (6.2)	n.r.	PCL-5	n.r.	n.r.	1 - 2x 3 cm, LIA	No associations with PTSD symptom severity
Schumacher *et al.*, 2022 [41]	Singular	PTSD (*n* = 19)	PTSD (*n* = 19)	Male adults, mixed age (Germany)	38.53 (10.23)	0	26.50 (3.61)	Deployment-related trauma	CAPS-5	n.r.	Only for most severe traumatic event: PTSD: 9.53 (6.79), TE: 8.89 (7.05)	1.5 cm, CLIA	No associations with PTSD symptom severity
		TE (*n* = 10)	TE (*n* = 10)		40.90 (9.47)	0	25.43 (1.90)						
		NTE (*n* = 10)	NTE (*n* = 10)		27.60 (4.03)	0	25.30 (1.97)						
Yirmiya *et al.*, 2022 [42]	Singular	78 samples (not specified whether TE/PTSD or NTE)	78 samples (not specified whether TE/PTSD or NTE)	Female adults, mixed age (Israel)	ca. 38 (n.r.)	100	n.r.	mixed	PCL-5	n.r.	n.r.	3 cm, ELISA	Positive associations with trauma exposure

**Notes:** CA = childhood abuse; CAPS = Clinically-Administered PTSD Scale [66]; CAPS-5 = Clinically-Administered PTSD Scale for DSM-5 [67]; CBM-APP = Cognitive Bias Modification Training for Appraisals; CLIA = chemiluminescence immunoassay; d = days; DIA-X/M-CIDI = Diagnostisches Expertensystem für Psychische Störungen [68]/ Composite International Diagnostic Interview – Military [69]; ELISA = Enzyme-linked immunosorbent assay; HCC = hair cortisol concentration; HR-MS/MS = high resolution tandem mass spectrometry; HTQ = Harvard Trauma Questionnaire [70]; IES-R = Impact of Events Scale – Revised [84]; IPV = intimate partner violence; IQR = interquartile range; LC-MS/MS = liquid chromatography tandem mass spectrometry; LEC = Life Event Checklist [85]; LEC-5 = Life Event Checklist for DSM-5 [71]; LIA = Luminescent Immunoassay; LRA = Lord’s Resistance Army; LSC-R = Life Stressor Checklist – Revised [72]; LTE = lifetime trauma exposure; M = mean; m = months; MDD = major depressive disorder; Med = median; n = number of participants; n.r. = not reported; NTE = non-trauma-exposed participants; PCL-5 = PTSD Checklist for DSM-5 [73]; PCL-C = PTSD Checklist – Civilian Version [74]; PC-PTSD = Primary Care PTSD Screen [76]; PDS = Posttraumatic Diagnostic Scale [77]; PLES = Police Life Events Checklist [78]; PSS-I = PTSD Symptom Scale Interview; PSS-SR = PTSD Symptom Scale – Self Report [79]; PTCI = Postttraumatic Cognitions Inventory [86]; PTSD = posttraumatic stress disorder; RESQ-CE = Rescue and Emergency Situations Questionnaire – Critical Exposure [87]; SCID-I = Structured Clinical Interview for DSM-IV [80]; SD = standard deviation; t0 = first assessment timepoint, t1 = second assessment timepoint, t2 = third assessment timepoint; TE = trauma-exposed participants who did not fulfil the criteria for a PTSD diagnosis; THQ = Trauma History Questionnaire [81]; TSC-40 = Trauma Symptom Checklist – 40 [88]; w = weeks; WEQ = War-Exposure Questionnaire [82]; y = years.

**Table 3:** Overview of studies on HCC as a prognostic biomarker in trauma/PTSD.

**Author (Year)**	**Assessment Times**	**Sample**	**Sample for HCC Analyses**	**Brief Sample Description, Country**	**Age** **(*M, SD*)**	**Sex (% Female)**	**BMI** **(*M, SD*)**	**Type of Focus Trauma**	**Assessment of PTSD**	**Assessment of LTE**	**Timing of LTE**	**Hair Segments, Analysis Method**	**Results with Respect to HCC as a Prognostic Biomarker**	**Further Variables Included in the Respective Models**
Steudte-Schmiedgen *et al.*, 2015 [46]	t0 (before deployment), t1 (12 m after deployment), HCC at t0 and t1	TE/PTSD (*n* = 113)	TE/PTSD (*n* = 113)	Young, male soldiers (Germany)	27.68 (6.11)	0	25.45 (2.69)	combat & civilian	DIA-X/ M-CIDI; PCL-C	Yes, DIA-X/ M-CIDI	n.r. for baseline LTE	2 cm, LC-MS/ MS	↓: Lower HCC at t0 predictive for higher PTSD symptom increase at t1 upon trauma exposure	t0 PTSD symptoms, number of t0 LTE
		NTE (*n* = 129)	NTE (*n* = 129)											
Pacella *et al.*, 2017 [60]	t0 (30d post-injury), t1 (60d post-injury), HCC only at t0	TE (*n* = 34)	TE (*n* = 30)	Adults, mixed age/gender (U.S.)	33.1 (12.9)	71	n.r.	Physical injury following accidents or assault	PDS, PCL-C	Yes, PDS	only for injury (30 /60 days)	3 cm, LC-MS/ MS	↑: Higher HCC at t0 predictive of higher avoidance, numbing, and overall PTSD symptoms at t1	t0 PTSD symptoms, age, sex
Petrowski *et al.*, 2020 [55]	t0 (within first 10d after motor vehicle crash), t1 (t0 + 3m), HCC at t0 and t1	Motor vehicle crash victims (*n* = 62)	Motor vehicle crash victims (*n* = 61)	Adults, mixed age/gender (Germany)	43.75 (13.51)	92.9	26.31 (5.46)	Motor vehicle crash	SCID-I, PDS, IES-R	Yes, exclusion of previous trauma *via* THQ, SCID-I	only for motor vehicle crash	3 cm, LC-MS/ MS	↑: Higher HCC at t0 predictive of higher avoidance behavior, but not any other symptom cluster at t1	t0 PTSD symptoms
Sopp *et al.*, 2021 [56]	t0, t1 (t0 + 6m), t2 (t0 + 12m), HCC only at t0	Firefighters (*n* = 529)	Firefighters (*n* = 371)	Adults, mixed age/gender (The Netherlands)	38.78 (10.10)	7.28	n.r.	Mixed, focus on work-related trauma	PCL-5	Yes, self-developed questionnaire on work-related trauma, LEC-5	n.r.	2 cm, n.r.	HCC at t0 not predictive of PTSD symptom severity at t1 or t2	LTE, y of service, type of service, psychopathology, sex, work-related trauma severity

**Notes:** d = days; DIA-X/M-CIDI = Diagnostisches Expertensystem für Psychische Störungen [68]/Composite International Diagnostic Interview – Military [69]; HCC = hair cortisol concentration; IES-R = Impact of Events Scale – Revised [84]; LC-MS/MS = liquid chromatography tandem mass spectrometry; LEC-5 = Life Event Checklist for DSM-5 [71]; LTE = lifetime trauma exposure; M = mean; m = months; n = number of participants; n.r. = not reported; PCL-C = PTSD Checklist – Civilian Version [74]; PCL-5 = PTSD Checklist for DSM-5 [73]; PDS = Posttraumatic Diagnostic Scale [77]; PTSD = posttraumatic stress disorder; SD = standard deviation; SCID-I = Structured Clinical Interview for DSM-IV [80]; t0 = first assessment timepoint, t1 = second assessment timepoint, t2 = third assessment timepoint; TE = trauma-exposed participants who did not fulfill the criteria for a PTSD diagnosis; THQ = Trauma History Questionnaire [81]; y = years.

**Table 4:** Overview of studies on HCC as an intervention-related biomarker in trauma/PTSD.

**Author (Year)**	**Assessment Times**	**Sample**	**Sample for HCC Analyses**	**Brief Sample Description, Country**	**Age (*M, SD*)**	**Sex (% Female)**	**BMI (*M, SD*)**	**Type of Focus Trauma**	**Assessment of PTSD**	**Assessment of LTE**	**Timing of LTE**	**Hair Segments, Analysis Method**	**Results with Respect to HCC as an Intervention-Related Biomarker**	**Further Variables Included in the Respective Models**
Hummel *et al.*, 2021 [63]	t0 (pre-treatment), t1 (t0 + *M* = 63.6, *SD* = 14.5 d duration of treatment, post-treatment), t2 (t1 + 5 m, follow-up)	PTSD (*n* = 52)	PTSD (t0: *n* = 43, t1: *n* = 38, t2: *n* = 23)	Female adults, mixed age (Germany)	41.60 (10.54)	100	27.31 (5.38)	Mixed	SCID-I, diagnostic interview with psychologists, PDS	Yes, PDS checklist	n.r.	3 cm, LC-MS/MS	Increase from t0 to t2, but not from t0 to t1 or t1 to t2	treatment duration, BMI
Woud *et al.*, 2021 [83]	t0 (pre-training), t1 (t0 + 6w, post-training), t2 (t1 + 3 m, follow-up)	CBM-APP (*n* = 39)	CBM-APP (t0: *n* = 32, t1: *n* = 30, t2: *n* = 23)	Adults, mixed age and gender (Germany)	42.41 (12.42)	92.3	n.r.	Mixed	CAPS-5, PTCI, PCL-5	Yes, CAPS-5/ LEC-5	n.r.	3 cm, LC-MS/MS	No changes of HCC over the intervention	BMI
		Control: Peripheral Vision Task (*n* = 41)	Control: Peripheral Vision Task (t0: *n* = 25, t1: *n* = 26, t2: *n* = 19)		39.05 (12.45)	82.9								

**Notes:** BMI = Body Mass Index; CAPS-5 = Clinically-Administered PTSD Scale for DSM-5 [67]; CBM-APP = Cognitive Bias Modification Training for Appraisals; LC-MS/MS = liquid chromatography tandem mass spectrometry; LEC-5 = Life Event Checklist for DSM-5 [71]; LTE = lifetime trauma exposure; M = mean; n = number of participants; n.r. = not reported; PCL-5 = PTSD Checklist for DSM-5 [73]; PDS = Posttraumatic Diagnostic Scale [77]; PTCI = Postttraumatic Cognitions Inventory [86]; PTSD = posttraumatic stress disorder; SD = standard deviation; SCID-I = Structured Clinical Interview for DSM-IV [80]; t0 = first assessment timepoint, t1 = second assessment timepoint, t2 = third assessment timepoint; w = weeks.

**Table 5:** Reporting standards and study quality of the included studies on HCC in the context of trauma/PTSD.

**Author (Year)**	**Participants’ Trait ** **Characteristics**		**Participants’ ** **Substance and Medication Intake**		**Participants’ Health-related Characteristics**		**Participants’ Hair Characteristics**		**Hair Sampling and Analysis Factors**		**Overall Score**	
Steudte *et al.*, 2011 [39]	+	0.600	++	0.875	+	0.625	=	0.500	=	0.455	+	**0.611**
Andersen *et al.*, 2013 [59]	+	0.600	--	0.000	-	0.375	--	0.000	=	0.455	-	**0.306**
Steudte *et al.*, 2013 [45]	+	0.600	+	0.750	+	0.750	++	1.000	+	0.636	+	**0.722**
Gao *et al.*, 2014 [43]	+	0.700	--	0.125	-	0.375	=	0.500	+	0.636	=	**0.458**
Steudte-Schmiedgen *et al.*, 2015 [46]	+	0.600	=	0.438	+	0.688	-	0.375	-	0.364	=	**0.486**
Groër *et al.*, 2016 [52]	++	0.800	--	0.188	--	0.125	--	0.000	-	0.273	-	**0.264**
Bluemke *et al.*, 2017 [31]	=	0.500	--	0.125	-	0.375	--	0.000	--	0.182	-	**0.236**
Boeckel, Viola, Daruy-Filho, Martinez, & Grassi-Oliveira, 2017 [48]	+	0.600	-	0.250	-	0.250	--	0.000	=	0.545	-	**0.361**
Fischer *et al.*, 2017 [65]	++	1.000	++	0.875	--	0.000	=	0.500	=	0.591	=	**0.569**
Mewes *et al.*, 2017 [49]	+	0.700	--	0.000	--	0.000	+	0.625	=	0.455	-	**0.306**
Morris, Abelson, Mielock, & Rao, 2017 [53]	+	0.600	=	0.500	+	0.750	++	1.000	=	0.455	+	**0.611**
Pacella *et al.*, 2017 [60]	+	0.600	--	0.125	--	0.000	=	0.500	=	0.455	-	**0.306**
Heller *et al.*, 2018 [50]	+	0.600	-	0.375	--	0.125	--	0.000	=	0.500	-	**0.347**
Schalinski, Teicher, & Rockstroh, 2019 [61]	++	0.800	=	0.563	-	0.375	+	0.625	=	0.545	=	**0.556**
Söder, Clamor, & Lincoln, 2019 [58]	++	1.000	+	0.625	=	0.500	--	0.000	-	0.364	=	**0.500**
van Zuiden *et al.*, 2019 [44]	++	0.800	+	0.625	-	0.250	+	0.750	+	0.636	=	**0.583**
Behnke *et al.*, 2020 [62]	++	0.800	--	0.125	-	0.250	+	0.750	-	0.364	-	**0.389**
Buchmüller *et al.*, 2020 [54]	+	0.700	--	0.125	=	0.438	+	0.750	-	0.364	=	**0.417**
Castro-Vale *et al.*, 2020 [47]	++	0.800	+	0.688	+	0.750	+	0.750	-	0.318	+	**0.611**
Petrowski *et al.*, 2020 [55]	+	0.600	=	0.438	+	0.625	--	0.000	-	0.364	=	**0.431**
van den Heuvel el al., 2020 [40]	++	1.000	=	0.500	=	0.500	+	0.750	=	0.591	+	**0.625**
Hummel *et al.*, 2021 [63]	+	0.600	=	0.438	-	0.375	+	0.750	-	0.273	=	**0.431**
Sopp *et al.*, 2021 [56]	-	0.300	--	0.000	--	0.000	--	0.000	-	0.273	--	**0.125**
Spikman *et al.*, 2021 [33]	+	0.600	--	0.125	--	0.125	--	0.000	=	0.409	-	**0.264**
Woud *et al.*, 2021 [83]	++	1.000	-	0.375	--	0.125	--	0.000	-	0.273	-	**0.333**
Basso *et al.*, 2022 [64]	++	0.800	=	0.438	-	0.250	+	0.750	+	0.727	=	**0.569**
Bob *et al.*, 2022 [57]	-	0.300	-	0.250	-	0.375	--	0.000	-	0.273	-	**0.264**
Lynch *et al.*, 2022 [51]	++	0.800	--	0.125	--	0.125	=	0.500	=	0.545	-	**0.389**
Marcil *et al.*, 2022 [32]	++	0.900	-	0.250	--	0.188	=	0.500	-	0.364	-	**0.389**
Schumacher *et al.*, 2022 [41]	++	0.800	-	0.313	=	0.500	+	0.625	+	0.636	=	**0.556**
Yirmiya *et al.*, 2022 [42]	+	0.600	--	0.000	--	0.000	--	0.000	=	0.591	-	**0.264**
**Mean Overall Score**	+	**0.700**	-	**0.343**	-	**0.329**	=	**0.403**	=	**0.449**	=	**0.428**
**SD Overall Score**		**0.179**		**0.258**		**0.241**		**0.352**		**0.141**		**0.146**

**Notes:** The criteria correspond to the following individual items: Trait characteristics of the participant (age, sex, body mass index, socioeconomic status, ethnicity). Substance and medication intake of the participant (nicotine, alcohol, drugs, hormonal contraceptives, overall, psychotropic, endocrine, specifically glucocorticoid-containing medication). Health-related characteristics of the participant (presence of severe/chronic physical or psychological conditions, specifically endocrine disorders, pregnancy, lactation/breastfeeding, menopause, major rhythm changes, subjectively experienced stress). Hair characteristics (natural color, curls/waves, washing frequency, hair treatments). 5) hair sampling and analysis factors (season of sampling, sampled at posterior vertex, length ≤ 6 cm, hair mass, storage time, analysis in one batch, inter- and intra-assay coefficients of variance, non-detectables and outliers, corrections for skewness). Ratings correspond to whether studies 0) did not; or 1) did report on a confounder; or 2) did control for it by demonstrating no respective group differences, calculating its association with HCC, adding it as a model covariate, or excluding it/fixing it to a certain value/imputing it. We calculated mean scores for each category (1: Five items corresponding to a range of 0-10; 2: eight items corresponding to a range of 0-16; 3: eight items corresponding to a range of 0-16; 4: four items corresponding to a range of 0-8; 5: 11 items corresponding to a range of 0-22). The resulting mean scores were then rated as – (x < 0.2, no to minimal reporting), - (0.2 ≤ x < 0.4, poor reporting), = (0.4 ≤ x < 0.6, average reporting), + (0.6 ≤ x < 0.8, good reporting), and ++ (0.8 ≤ x ≤ 1, very good to excellent reporting).

## Data Availability

The data supporting the findings of the article (Appendix A and B) are available in OSF at https://osf.io/7mdqz/?view_only=b8d04e71073f46ecbf34122cdfd83921.

## LIST OF ABBREVIATIONS

ACTH: Adrenocorticotropin-releasing Hormone
AVP: Arginine-vasopressin
CNS: Central Nervous System
CRH: Corticotropin-releasing Hormone
HCC: Hair Cortisol Concentration
HPA: Hypothalamic-pituitary-adrenal
LTE: Lifetime Trauma Exposure
NTE: Non-trauma-exposed
PTSD: Posttraumatic Stress Disorder
PVN: Paraventricularis
TE: Trauma-exposed

## STANDARDS OF REPORTING

PRISMA guidelines and methodology were followed.

## SUPPLEMENTARY MATERIAL

PRISMA checklist is available as supplementary material on the publisher’s website along with the published article.
